# A moral history of seizures: reported causes of seizures in the nineteenth century

**DOI:** 10.1055/s-0045-1806829

**Published:** 2025-06-01

**Authors:** Márcio Pinheiro Lima, Elza Márcia Targas Yacubian

**Affiliations:** 1 Universidade Federal de São Paulo, Escola Paulista de Medicina, Departamento de Neurologia, São Paulo SP, Brazil.

**Keywords:** Epilepsy, History, 19th Century, History of Medicine, Neurology

## Abstract

In the nineteenth century, neurology was in its infancy as an organized medical specialty. At that time, seizures were often attributed, under the guise of scientific explanations, to moral causes or behaviors. The medical literature from this period contains references to poor parental care as a cause of epilepsy and descriptions of seizures being inherited alongside other undesirable traits. Temperance was praised, while gluttony was associated with epileptic fits. Unwanted sexual behaviors, such as masturbation, were also considered a risk factor for seizures. Women were thought to be more prone to epilepsy due to their behaviors and emotional disposition. These accounts illustrate the interplay between neurological science and the prevailing social norms of the era.

## INTRODUCTION

The first known mention to epilepsy dates from ∼ 2000 BC, documented in an Akkadian tablet describing a person with “his neck turning left, hands and feet [...] tense, and his eyes wide open, and from his mouth froth is flowing without him having any consciousness.” 1 Nowadays, epilepsy is defined conceptually as “a disease of the brain characterized by an enduring predisposition to generate epileptic seizures.” 2 Throughout history, seizures have been attributed to various causes. By the nineteenth century, neurology had begun to emerge as an organized medical specialty, with notable figures such as Jean Martin-Charcot (1825–1893), Guillaume-Benjamin Duchenne (1806–1875), and Moritz Heinrich Romberg (1795–1873) contributing significantly to the field. However, the scientific literature from this period often linked seizures to undesirable or “antisocial” habits, frequently imbued with moral judgment.

## PARENTAL CARE AND HEREDITY

Seziures were frequently attributed to failures in child-rearing practices or to hereditary traits associated with parental habits deemed innapropriate. There is an account of two children developing seizures due to the parents' inattention to their proper feeding, 3 for instance, and claims that epileptic children might be cured by the “intelligent care and nursing of the parents.” 4 Consanguinity was often cited as a common risk factor linking drunkenness and epilepsy, both framed as inherited medical conditions. 5 Maternal care was subject to intense scrutiny, as illustrated in a case in which a child's epilepsy was attributed to “protracted lactation” by the mother. 6

## SEXUALITY

Deviations from the sexual norms of the time were frequently cited as plausible causes of epilepsy, often attributed to the purported excitatory effects of the genitals on the epileptic brain. 7 8 Such notions were so entrenched that in certain case reports 9 10 castration and circumcision were used as a treatment for epilepsy, with positive results. Masturbation was identified as a cause of neuronal hyperexcitability, an idea supported even by prominent figures such as Sir William Gowers (1845–1915; who, however, also suggested some of these events could be psychogenic). 10 The epidemiological studies of the time correlated masturbation and “episodes of libertinage” with epilepsy. 11 In one case, 12 a priest's epilepsy was attributed to celibacy and masturbation, with observations that celibacy conflicted with his “passion and social feelings,” and that similar cases had been described by François-Joseph-Victor Broussais (1772–1838), Jean-Alfred Fournier (1832–1914), and Samuel-August Tissot (1728–1797). Masturbation was further linked to headaches and other brain diseases, being labeled as an “evil” habit. Other urological conditions, such as phimosis, were similarly implicated in the development of epilepsy. 13

## TEMPERANCE AND GLUTTONY

Even the circumstances of conception could serve as justifications for epilepsy, particularly when coupled with socially-undesirable behaviors. One report 14 attributed a child's epilepsy to the father being intoxicated at the time of conception, a case cited to advocate for temperance. Gluttony was another behavior associated with epileptic fits, framed as a moral failing that could provoke or exacerbate the condition. 15

## EPILEPSY AND WOMANHOOD

**Figure 1:**
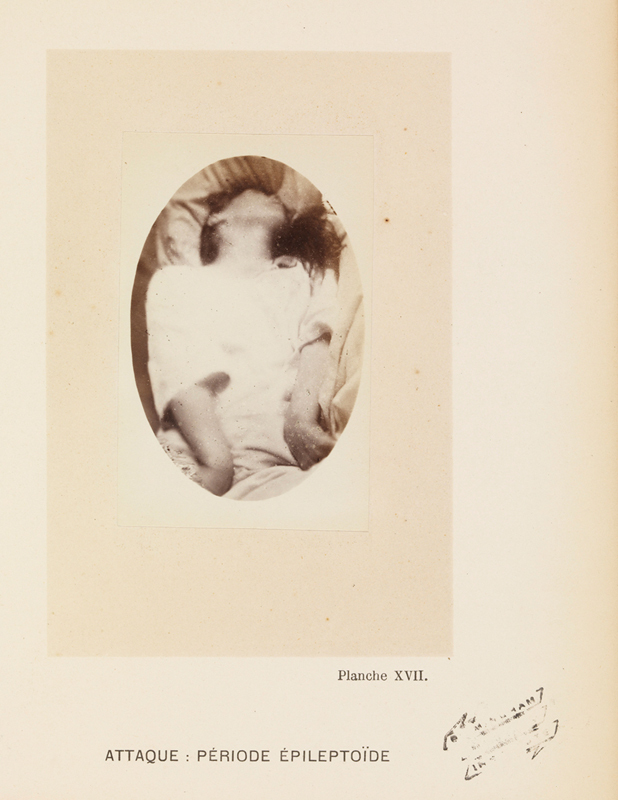
Photographic plate of a woman having a seizure at La Salpêtrière in the 1870s. Source: Originally found at Iconographie photographique de la Salpêtrière: service de M. Charcot / par Bourneville et P. Regnard. This work is in public domain, with no copyright restrictions. Available at: https://wellcomecollection.org/works/r6mngdsr .

The medical literature of the nineteenth century frequently posited an innate predisposition among women to seizures, often explained through sociobiological characteristics. This predisposition was believed to be hereditary, with the children of “hysterical” mothers thought to be at higher risk of developing epilepsy. 16 17 One account 18 suggested that female clothing, described as excessively heavy and exerting pressure on the uterus, could cause reflex seizures. Women with epilepsy were often photographed to serve as teaching aids, as exemplified in Figure 1 .

The reports herein discussed contextualize the early development of neurology within the social and historical framework of the nineteenth century, highlighting how societal ideals and moral judgments influenced scientific explanations of biological phenomena. These examples not only provide insights into the neurological ideas of the time, but also serve as a reminder of how social context can shape medical practice and thinking.
